# Two Mistaken Beliefs about Suicide

**Published:** 2019-04

**Authors:** Saxby Pridmore, Jamshid Ahmadi, William Pridmore

**Affiliations:** 1 Department of Psychiatry, University of Tasmania, Hobart, Tasmania, Australia.; 2 Substance Abuse Research Center, Shiraz University of Medical Sciences, Shiraz, Iran.; 3 School of Medicine, Australian National University, Canberra, Australia.

Suicide has been known in all cultures, every region and ethnic group. Throughout history, the first recorded suicides were committed by Pyramus and Thisbe, who were lovers that died in Babylonia, Persia, around 2000 BC. 

In 2016, the highest suicide rate in the world belonged to Guyana (30/100 000 p.a.), followed by Lesotho and Russia. Kazakhstan, an Asian country, filled the sevenths place (22.8/100 000 p.a.).

For more than a millennium, Islam and Christianity considered suicide to be a sinful practice. In the West, in the early 19th Century, the belief that suicide was a religious problem was replaced by the belief that suicide was a medical problem and could be prevented by treatment. In 2014, WHO described that belief as a “myth” (1); however, studies continued to focus on mental disorders as the primary etiological factors and experts recommend treatment for its prevention.

A related belief is that suicide risk factors can be managed by health professionals, and thereby all those people about to commit suicide can be identified with certainty (2). It is also assumed that once a high-risk individual has been identified, suicide prevention is assured.

Are mental disorders the sole trigger of suicide? 

Mental disorders are painful, and there is no doubt that people with mental disorders complete suicide more often than those without mental disorders. However, many people without mental disorders also complete suicide (1).

Initially, the belief that suicide is always triggered by a mental disorder was thought to be supported by the findings of psychological autopsies. However, recently, this method has been described as “theoretically, methodologically, and analytically flawed” (3) and an approach that should now be abandoned (4).

Part of the problem is that mental disorder has not been clearly defined. Existing definitions do not differentiate disorders from the distress encountered in everyday life. Thus,whenever a suicide is associated with distress (presumably every case), it can be interpreted as being caused by mental disorder.

Our group has proposed a predicament model of suicide, in which suicide is conceptualized as a response to unpleasant predicaments from which there are limited escape options (5). In this model, mental disorder represents a serious predicament, but others include a wide range of social, economic, cultural, and physical health challenges.

What are suicide risk factors? 

Epidemiological and psychological autopsy studies have identified many variables associated with suicide, including mental disorder, male gender, single marital status, alcohol use, physical health problems, and unemployment. It was believed that the list of suicide risk factors could be made so comprehensive that all individuals at high risk (who would complete suicide) could be identified and that treatment of such persons would prevent suicide (6). 

Recent systematic reviews (7) and meta-analyses (8) have concluded that after 40 years of research, no list has been produced which identifies all those who will suicide. This is not surprising given that suicide is a rare event that 95% of those who are designated as high-risk do not die by suicide, and 50% of those who do die by suicide come from the group which would be classified as low-risk. The use of such risk-factor lists is no longer supported. It is argued the time spent on completing suicide risk factor assessments is time which should be spent providing clinical care.

A consequence of the incorrect belief that all suicides are due to a mental disorder was that the responsibility for preventing suicide was placed exclusively with medical profession. However, in addition to mental disorder, completed suicide has roots in a wide range of cultural, economic, educational, family, and social circumstances. Thus, suicide is often well outside the resources of medicine and would usually be more appropriately classified as a community problem. The focus on mental disorder as “the trigger” has silenced public discussion and discouraged participation by those with influence in a range of relevant fields.

The incorrect belief that risk factors can identify all those who will complete suicide and that appropriate treatment will prevent this outcome has placed mental health professionals in unfair and impossible situations. In the event of suicide by a person who has not been admitted to hospital, the doctor is criticized for not having arranged admission. In the event of suicide by a person who has been admitted to hospital, the doctor is criticized for not having predicted the suicide and not taking all necessary steps. It must be remembered that even when suicide by a particular individual incarcerated in highly specialized facilities is considered likely and all recommended actions are taken, suicide remains possible.

For the sake of both citizens and the medical profession, these incorrect beliefs must be rejected.
